# Analyzing the causal relationship between gut microbiotas, blood metabolites, and COVID-19 susceptibility: A Mendelian randomization study

**DOI:** 10.1097/MD.0000000000041445

**Published:** 2025-04-04

**Authors:** Xiao-Yan Yao, Yan-Hua Zhang, Yu-Wei Weng, Jian-Feng Xie, Kui‐Cheng Zheng

**Affiliations:** a The School of Public Health, Fujian Medical University, Fuzhou, China; b Fujian Provincial Center for Disease Control and Prevention, Fujian Provincial Key Laboratory of Zoonosis Research, Fuzhou, China.

**Keywords:** blood metabolites, COVID-19, gut microbiotas, mediating effects, Mendelian randomization analysis

## Abstract

Gut microbiota and blood metabolites play crucial roles in the progression and outcomes of COVID-19, but the causal relationships and mechanisms remain unclear. Our aim is to use two-sample Mendelian randomization (MR) to explore the causal relationships between gut microbiota, COVID-19 susceptibility, and potential mediating blood metabolites. We utilized summary statistics from the largest genome-wide association studies (GWAS) to date on gut microbiota (n = 18,340), blood metabolites (n = 115,078), and COVID-19 susceptibility (cases n = 60,176 and controls n = 1310,725 from the COVID-19 Host Genetics Initiative meta-analysis). We conducted bidirectional MR analyses to explore the causal relationships between gut microbiota and COVID-19 susceptibility and performed two-step MR to identify potential mediating blood metabolites. Five analytical methods were used to assess two-sample causal relationships, with inverse variance weighted (IVW) being the primary method. Sensitivity analyses were also conducted to ensure the robustness of the main MR results. Using the IVW method, we found causal relationships between 3 types of gut microbiota and 34 blood metabolites with COVID-19 susceptibility. In the two-step MR, the non-oxidative branch of the Pentose phosphate pathway was shown to reduce Sebacate (C10-DC) levels, and the species *Parabacteroides goldsteinii* was negatively correlated with Acetoacetate levels. Sebacate (C10-DC) levels were negatively associated with COVID-19 susceptibility, while Acetoacetate levels were positively associated with COVID-19 susceptibility. Furthermore, these causal relationships remained significant after correcting for false discovery rates (all q-values < 0.05). Heterogeneity and pleiotropy tests showed no statistical significance (*P* > .05). Mediation analysis indicated that the abundance of the non-oxidative branch of the Pentose phosphate pathway and COVID-19 susceptibility was mediated by Sebacate (C10-DC) levels (mediation proportion of 15.8%), and the abundance of *P goldsteinii* and COVID-19 susceptibility was mediated by Acetoacetate levels (mediation proportion of 31.7%). The current MR study provides evidence supporting the causal relationships between several specific gut microbiotas and COVID-19 susceptibility, as well as potential mediating blood metabolites. Our findings warrant further validation through larger epidemiological studies.

## 1. Introduction

Corona Virus Disease 2019 (COVID-19), a contagious disease, has spread worldwide and primarily affects the respiratory system.^[1]^ Most COVID-19 patients exhibit mild symptoms with a good prognosis.^[2]^ Unfortunately, some patients may rapidly progress to severe syndromes such as pneumonia, acute respiratory distress syndrome (ARDS), and multiple organ failure, leading to death.^[3]^ One of the most crucial methods to prevent COVID-19 is to explore its biomarkers and identify potential risk factors.

Changes in human gut microbiota and blood metabolite are closely associated with various diseases, playing significant roles in regulating metabolism, immunity, digestion, and absorption. Substantial evidence suggests that gut microbiota play a critical role in the progression and outcomes of COVID-19.^[4]^ Zhang et al noted impaired L-isoleucine biosynthesis capabilities in the gut microbiota of COVID-19 patients, which persist even after disease resolution.^[5]^ Multiple clinical studies have also shown significant changes in blood metabolites in COVID-19 patients, potentially mechanisms underlying excessive inflammation, predominantly involving amino acid and lipid metabolic pathways.^[6–8]^ Recent research has linked changes in gut microbiota and blood metabolite concentrations to COVID-19. Studies indicate that during the recovery process from COVID-19, both oral and gut microbiome diversity gradually increase, accompanied by significant changes in blood metabolite abundance.^[9]^ However, the underlying causal relationships remain unclear. Multiple transcriptomic studies indicate significant interactions between gut microbiota and blood metabolites. Therefore, this study hypothesizes that blood metabolites may mediate the impact of gut microbiota on susceptibility to COVID-19.

Mendelian randomization (MR) is an analytical method that uses genetic variations as instrumental variables to explore causal relationships between exposures and outcomes.^[10]^ It helps reduce confounding effects and avoids reverse causation. Previous MR studies have explored causal relationships between blood metabolites, gut microbiotas, and COVID-19, but they have not further investigated the causal relationships between gut microbiotas and blood metabolites in COVID-19 patients, nor focused on the mediating effects of blood metabolites.^[11,12]^ Hence, Our study applied MR methods to analyze the relationships between 1400 blood metabolites, 412 gut microbiotas, and susceptibility to COVID-19, with a focus on the mediating effects of blood metabolites.

## 2. Methods

### 2.1. Study design

**Figure 1.**
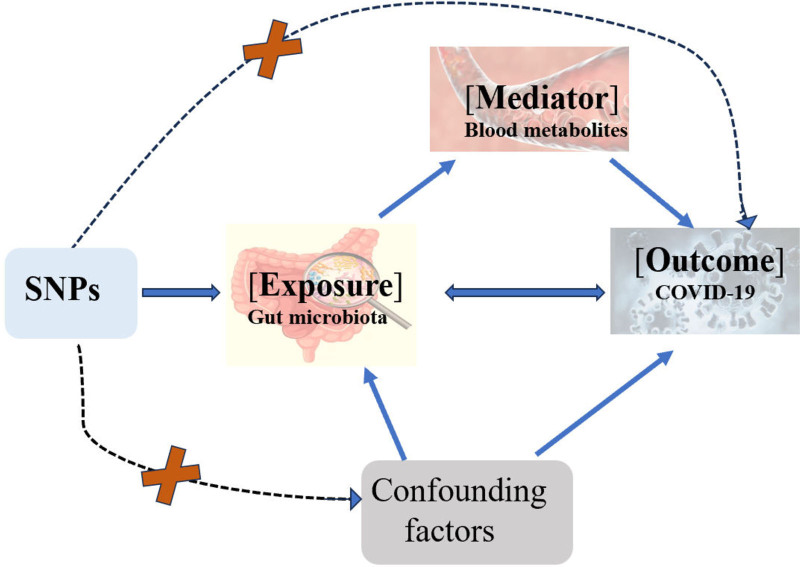
That illustrates the assumptions and design of the bidirectional and mediation Mendelian randomization (MR) analyses.

According to the STROBEMR statement, to ensure the validity of the results, the instrumental variables need to meet 3 assumptions.^[13]^ First, the instrumental variables must be strongly associated with the exposure factor. Second, the genetic variations used for the exposure factor must be independent of any confounding variables. Finally, it is assumed that the instrumental variables affect the outcome solely through the exposure factor and not through other pathways. In this study, the gut microbiotas and blood metabolites were used as exposure factors, and significantly associated single nucleotide polymorphisms (SNPs) were selected as IVs, with COVID-19 susceptibility as the outcome variable. The research process is shown in Figure 1 (see Fig. 1, Which illustrates the Assumptions and design of the bidirectional and mediation Mendelian randomization analyses).

### 2.2. Data sources

The GWAS summary data used in this study are publicly available online. The primary source is a GWAS summary data website (https://www.ebi.ac.uk/gwas/), which aggregates multiple GWAS datasets. The GWAS summary data include information such as SNPs, chromosome and specific positions, encoded genes, effect sizes, effect *P* values, and sample sizes.^[14]^ Details about the data sources and sample sizes used in this study are summarized in Supplemental Table S1 (see Table S1, Supplemental Digital Content, http://links.lww.com/MD/O434, Which illustrates the characteristics of summary association studies). All relevant studies have received ethical approval. To make the analysis more comprehensive, our study utilized the existing GWAS dataset with the greatest variety of gut microbiota and blood metabolites.

### 2.3. Gut microbiota

GWAS summary statistics related to gut microbiotas were collected from a genome-wide association study in the Netherlands.^[15]^ The summary statistics for each gut microbiota can be publicly accessed from the GWAS catalog (accession numbers GCST90027446 to GCST90027857). This includes 207 microbial taxa (5 phyla, 10 classes, 13 orders, 26 families, 48 genera, and 105 species) and 205 functional pathways.^[15]^ The study is based on the multi-disciplinary prospective LifeLines cohort, which includes 8719 participants with complete stool and phenotype information. After strict quality control, the final sample size was 7738, including 5584,686 SNPs.

### 2.4. Blood metabolite data

The blood metabolite data include 1091 metabolites and 309 metabolite ratios from 8299 individuals in the Canadian Longitudinal Study on Aging (CLSA) cohort.^[16]^ These metabolites span multiple metabolic pathways and have been linked to mechanisms of various diseases, including lipoprotein lipids, fatty acids, and fatty acid composition, as well as low molecular weight metabolites such as amino acids, ketone bodies, and glycolysis metabolites quantified in molar concentration units.^[16]^ The GWAS accession numbers are GCST90199621-902010209.

### 2.5. Disease data

The data related to COVID-19 susceptibility were sourced from the COVID-19 Host Genetics Initiative GWAS meta-analysis Round 5 (https://www.covid19hg.org/results/r5/).^[17,18]^ we selected individuals of European ancestry to adhere strictly to research standards. COVID-19 susceptibility was defined as a positive reverse transcription-quantitative polymerase chain reaction (RT-qPCR) test for COVID-19, or a clinical diagnosis based on symptoms, epidemiology, and ICD codes (n = 29,071). The control group (n = 1559,712) included individuals who tested negative or did not meet the diagnostic criteria for COVID-19.^[19]^ The GWAS accession number is GCST011071.

### 2.6. Data analysis

In the forward MR analysis, we selected SNPs associated with microbiota and blood metabolites as eligible instrumental variables (Ivs) based on the following criteria: SNPs significantly associated with gut microbiotas and blood metabolites (*P* ≤ 1 × 10^−5^). Linkage disequilibrium for significant SNPs related to exposure must meet *r*^2^ < 0.001 and genetic distance of 5000 kb. Exclusion of allelic inconsistent SNPs (e.g., A/G vs A/C). Removal of palindromic SNPs with ambiguous alleles (e.g., A/T or G/C) before data harmonization. MR-PRESSO test for pleiotropy and outliers with *P* > .05.^[20]^

We calculated the F-statistic for each IV to assess their strength using the formula. F > 10 indicates no significant weak instrument bias, defining the eligible SNPs.^[21]^ Harmonization of SNPs between the GWAS data for gut microbiota or blood metabolites and COVID-19 susceptibility ensured alignment of effect alleles. Due to the inability of this study to correctly distinguish the direction of causality, there is a possibility of reverse causality between the gut microbiota or blood metabolites and COVID-19. Reverse MR analysis is needed in this study to rule this out. In the reverse MR analysis, we used the same threshold (*P* < 1 × 10^−5^) to select IVs for COVID-19 susceptibility phenotypes and applied the same criteria to identify eligible IVs.

We conducted bidirectional Mendelian analyses to explore the causal relationships between gut microbiotas, blood metabolites, and COVID-19 susceptibility. MR methods such as inverse variance weighted (IVW), MR-Egger regression, weighted median (WME), and weighted mode were employed to test for causal relationships.^[22]^ Since the pleiotropy test in this study was not statistically significant, IVW was ultimately used for the MR analysis, with the other methods being used as supplementary. The IVW method reports the β-values for continuous outcomes and odds ratios (OR) with 95% confidence intervals (CI) for binary outcomes.^[23]^ *P *< .05 were considered statistically significant. The IVW method uses random effects to meta-analyze SNP-specific Wald estimates (SNP outcome estimate divided by SNP exposure estimate) to obtain the final causal effect estimate. The false discovery rate (FDR) method was used for multiple testing correction, providing FDR q-values.^[24]^ Associations with *P *< .05 but FDR ≥ 0.1 were considered suggestive, FDR < 0.1 indicated a positive association.

For the mediation analysis of “gut microbiotas – blood metabolites – COVID-19 susceptibility,” we used the two-step Mendelian randomization (TSMR) method to partition the direct and indirect effects of gut microbiotas and blood metabolites on COVID-19 susceptibility. TSMR assumes no interaction between exposure and mediator.^[25]^ First, we obtained the basic effect estimate of exposure (gut microbiota) on the outcome (COVID-19) (β0). Second, we performed two-step MR analysis to detect potential mediating metabolites: Calculating the causal effect of exposure (gut microbiota) on the mediator (blood metabolites) (β1). Calculating the causal effect of the mediator (blood metabolites) on the outcome (COVID-19) (β2), ensuring that the IVs used in the second step were not the same as those used in the first step. The causal effect of gut microbiota on COVID-19 susceptibility mediated by blood metabolites was estimated using the coefficient product method (β1×β2). The mediation proportion was calculated as “indirect effect/total effect” ([β1×β2]/β0).^[26]^

### 2.7. Sensitivity analysis

To further verify the stability and reliability of the results, we performed quality control, sensitivity analysis, and tests for heterogeneity and pleiotropy. Sensitivity analysis using the leave-one-out method assessed the influence of each SNP on the overall result by iteratively removing each SNP and calculating the results for the remaining SNPs. If no significant differences were observed, it indicated that the specific SNPs did not nonspecifically influence the effect estimate. Cochran’s Q test quantified heterogeneity among SNPs, with *P* > .05 indicating no heterogeneity evidence, suggesting that the fixed-effect IVW method was appropriate; otherwise, the random-effect IVW method was used.^[27]^ MR-Egger’s and intercept were used to assess potential heterogeneity and horizontal pleiotropy.^[27]^ MR-PRESSO analysis was used to remove significant outliers and reduce horizontal pleiotropy.^[27]^ OR and 95% CIs were used to evaluate the causal relationship in the two-sample MR analysis.

All MR analyses were conducted using R (version 4.3.3; R Foundation for Statistical Computing, Vienna, Austria), employing packages such as “TwoSampleMR,” “tidyverse,” “ggplot2,” “purrr,” and “readxl.” The “p.adjust” function in R estimated FDR_q values. MR results were visualized using scatter plots, forest plots, leave-one-out plots, and funnel plots.

## 3. Results

### 3.1. Selection of exposure instrumental variables

After filtering, the final number of SNPs used for the analysis related to 412 gut microbiotas and pathways ranged from 1 to 20 (median, 10). For 1400 metabolites levels, the number of SNPs ranged from 12 to 93 (median, 24), and for COVID-19 susceptibility, 37 SNPs were included. The median F-statistic for gut microbiota was 28.195 (range: 19.028–330.904), for metabolites was 21.204 (range: 19.503–1981.160), and for COVID-19 susceptibility was 21.269 (range: 18.118–100.264) (see Tables S2–S4, Supplemental Digital Content, http://links.lww.com/MD/O434 illustrates the used instrumental variables for 412 gut microbiotas from EBI. Table S3, Supplemental Digital Content, http://links.lww.com/MD/O434 illustrates the used instrumental variables for 1400 blood metabolites from EBI. Table S4, Supplemental Digital Content, http://links.lww.com/MD/O434 illustrates the used instrumental variables for COVID-19 from EBI). F > 10 is considered sufficient for providing informative MR analysis.

### 3.2. Genetic causality and correlation between gut microbiotas and COVID-19 susceptibility

TSMR analysis was conducted on the selected SNPs for gut microbiota and COVID-19 susceptibility, with complete results provided in Table S5 (see Table S5, Supplemental Digital Content, http://links.lww.com/MD/O434, Which illustrates the 5 Mendelian randomization models estimate the causal effects of gut microbiotas on COVID-19). Using the IVW method, 3 gut microbiota taxa and 2 functional pathways were found to be associated with COVID-19 susceptibility, with FDR-corrected results indicating statistical significance (see Table 1 and Table S6, Supplemental Digital Content, http://links.lww.com/MD/O434, Table 1 illustrates the bidirectional Mendelian randomization analyses of the causal effects between gut microbiota and COVID-19. Table S6, Supplemental Digital Content, http://links.lww.com/MD/O434, Which illustrates the 5 bidirectional Mendelian randomization models estimate the causal effects of gut microbiotas on COVID-19). To assess bidirectional causality between gut microbiotas and COVID-19 susceptibility, reverse MR analysis was also performed on taxa showing causal relationships in the forward MR analysis. Results indicated that the Coriobacteriaceae family and Coriobacteriales order were associated with COVID-19 susceptibility (RE *P* < .05) (Table 1 and Table S6, Supplemental Digital Content, http://links.lww.com/MD/O434). Ultimately, 3 gut microbiota taxa significantly associated with COVID-19 susceptibility were included as IVs to evaluate their causal relationship. The study results indicated that increased abundance of the species *P goldsteinii* increased the risk of COVID-19 susceptibility (OR: 1.045, 95% CI: 1.000–1.092, *P* = .047, FDR = 0.047), while increased abundance of the Pentose phosphate pathway non-oxidative branch (OR: 0.912, 95% CI: 0.835–0.995, *P* = .039, FDR = 0.047) and L-isoleucine biosynthesis pathway (OR: 0.900, 95% CI: 0.829–0.977, *P *= .011, FDR = 0.019) decreased the risk of COVID-19 susceptibility (see Table 1, Which illustrates the bidirectional Mendelian randomization analyses of the causal effects between gut microbiota and COVID-19).

**Table 1:** Bidirectional Mendelian randomization analyses of the causal effects between gut microbiota and COVID-19.

Exposure	Outcome	Method	Nsnp	OR (95% CI)	*P*	RE_*P*	Q (*P*)	Egger (*P*)
Pentose phosphate pathway non oxidative branch	COVID-19	IVW	7	0.912 (0.835, 0.995)	.039	0.472	4.121 (0.532)	0.018 (0.429)
L-Isoleucine biosynthesis IV	COVID-19	IVW	9	0.900 (0.829, 0.977)	.011	0.955	4.564 (0.713)	0.031 (0.147)
f_Coriobacteriaceae	COVID-19	IVW	9	1.383 (1.127, 1.697)	.002	0.041	48.070 (0.000)	−0.028 (0.662)
o_Coriobacteriales	COVID-19	IVW	9	1.383 (1.127, 1.697)	.002	0.041	48.084 (0.000)	−0.027 (0.663)
s_Parabacteroides_goldsteinii	COVID-19	IVW	7	1.045 (1.000, 1.092)	.048	0.283	4.025 (0.546)	−0.039 (0.263)

Q(p), Cochran’s Q test quantified heterogeneity among SNPs, with *P* > .05 indicating no heterogeneity evidence; Egger (*P*), MR-Egger’s and intercept were used to assess potential heterogeneity and horizontal pleiotropy, with *P* > .05 indicating no pleiotropy evidence.

IVW = inverse variance weighted, Nsnp = number of single nucleotide polymorphism, OR = odds ratio, RE_p = reverse MR result.

The sensitivity analysis results showed that the direction of all Beta values across different methods was consistent, only the IVW method yielded significant results, indicating the findings are relatively unstable (see Table 1 and Figure S1, Supplemental Digital Content, http://links.lww.com/MD/O435, Table 1 illustrates the bidirectional Mendelian randomization analyses of the causal effects between gut microbiota and COVID-19. Figure S1, Supplemental Digital Content, http://links.lww.com/MD/O435, illustrates the scatter plots of the SNP- gut microbiotas and SNP-COVID-19 association estimates for 3 gut microbiotas). Heterogeneity and pleiotropy tests for *P goldsteinii*, Pentose phosphate pathway non-oxidative branch, and L-isoleucine biosynthesis pathway were not statistically significant (*P* > .05), suggesting that heterogeneity and pleiotropy do not influence the results (see Table 1 and Figure S2, Supplemental Digital Content, http://links.lww.com/MD/O435, Table1 illustrates the bidirectional Mendelian randomization analyses of the causal effects between gut microbiota and COVID-19. Figure S2, Supplemental Digital Content, http://links.lww.com/MD/O435, illustrates the Funnel plots between 3 gut microbiota and COVID-19 risk estimates). The “leave-one-out” analysis indicated that the effect sizes of the included IVs were close to the total effect size, with no SNPs significantly influencing the causal estimates (see Figure S3, Supplemental Digital Content, http://links.lww.com/MD/O435, Which illustrates the Leave-one-out plots showed sensitivity analysis results between 3 gut microbiotas and COVID-19 risk estimates). A forest plot was also used to visually display the estimated effect of each SNP on the influence of gut microbiota on COVID-19 susceptibility (see Figure S4, Supplemental Digital Content, http://links.lww.com/MD/O435, Which illustrates the Forest plots showed the association between 3 gut microbiotas and COVID-19 risk under the IVW method).

### 3.3. Genetic causality and correlation between blood metabolites and COVID-19 susceptibility

In assessing the causal relationship between 1400 blood metabolites and COVID-19 susceptibility, the complete MR results are shown in Table S7 (see Table S7, Supplemental Digital Content, http://links.lww.com/MD/O434, Which illustrates 5 Mendelian randomization models estimate the causal effects of blood metabolites on COVID-19). Using the IVW method, 38 metabolites were found to have statistically significant causal relationships with COVID-19 susceptibility (see Table S8, Supplemental Digital Content, http://links.lww.com/MD/O434, Which illustrates 5 bidirectional Mendelian randomization models estimate the causal effects of blood metabolites on COVID-19). Reverse MR analysis evaluating the causal effect of COVID-19 susceptibility on metabolites indicated that levels of Alpha-hydroxyisovalerate, Bilirubin degradation product, C16H18N2O5 (2), X-12847, and the Adenosine 5’-diphosphate (ADP) to valine ratio decreased following COVID-19 infection (*P* < .05) (see Table S8, Supplemental Digital Content, http://links.lww.com/MD/O434, Which illustrates 5 bidirectional Mendelian randomization models estimate the causal effects of blood metabolites on COVID-19).

Among the remaining 34 metabolites, 18 belonged to 4 known superpathways (lipids, amino acids, nucleotides, and energy); 5 were categorized as xenobiotics; 2 were classified as metabolite ratios; and the remaining 9 were classified as unknown molecules. Of the 18 known metabolites, Acetoacetate had the most significant negative impact on COVID-19 susceptibility (OR = 0.920, 95% CI: 0.859–0.984, *P* = .016, FDR = 0.036), while Sebacate (C10-DC) had the most significant positive impact (OR = 1.093, 95% CI: 1.027–1.162, *P* = .005, FDR = 0.030) (see Table 2, which illustrates the bidirectional Mendelian randomization analyses of the causal effects between blood metabolites and COVID-19).

**Table 2:** Bidirectional Mendelian randomization analyses of the causal effects between blood metabolites and COVID-19.

Exposure	Outcome	Method	Nsnp	OR (95% CI)	*P*	RE_*P*	Q (*P*)	Egger (*P*)
3-methyl-2-oxovalerate	COVID-19	IVW	14	0.928 (0.864, 0.997)	.041	0.707	7.722 (0.861)	0.007 (0.544)
Indolelactate	COVID-19	IVW	27	0.922 (0.877, 0.969)	.001	0.351	21.280 (0.727)	−0.001 (0.910)
Phenyllactate (PLA) in elite athletes	COVID-19	IVW	26	0.946 (0.905, 0.99)	.017	0.371	22.553 (0.604)	0.003 (0.534)
1-oleoylglycerol (18:1)	COVID-19	IVW	30	0.955 (0.912, 1.000)	.048	0.892	25.541 (0.65)	0.005 (0.554)
3-hydroxyoctanoate	COVID-19	IVW	20	1.081 (1.021, 1.146)	.008	0.653	16.392 (0.631)	0.008 (0.376)
2-hydroxyoctanoate	COVID-19	IVW	17	1.085 (1.029, 1.144)	.003	0.091	6.234 (0.985)	0.003 (0.729)
1-methyl-4-imidazoleacetate	COVID-19	IVW	25	0.952 (0.916, 0.989)	.012	0.205	18.764 (0.764)	0.008 (0.172)
Sebacate (C10-DC)	COVID-19	IVW	16	1.093 (1.027, 1.162)	.005	0.610	9.694 (0.839)	0.002 (0.873)
Gamma-glutamylmethionine	COVID-19	IVW	26	0.942 (0.890, 0.997)	.039	0.246	28.291 (0.295)	−0.004 (0.572)
Glutamine degradant	COVID-19	IVW	25	0.935 (0.890, 0.982)	.007	0.457	24.381 (0.44)	0.004 (0.450)
Isobutyrylglycine	COVID-19	IVW	27	1.049 (1.002, 1.098)	.04	0.888	31.903 (0.196)	−0.004 (0.621)
Palmitoyl dihydrosphingomyelin	COVID-19	IVW	31	0.947 (0.902, 0.994)	.027	0.809	32.936 (0.325)	−0.002 (0.744)
N-acetyl leucine	COVID-19	IVW	18	0.932 (0.876, 0.991)	.026	0.324	6.904 (0.985)	0.003 (0.748)
Allantoin	COVID-19	IVW	22	1.081 (1.014, 1.153)	.017	0.111	26.065 (0.204)	0.000 (0.967)
Acetoacetate	COVID-19	IVW	13	0.920 (0.859, 0.984)	.016	0.522	12.58 (0.400)	−0.006 (0.538)
Linoleate (18:2n6)	COVID-19	IVW	20	0.938 (0.881, 0.999)	.046	0.142	9.637 (0.961)	0.004 (0.698)
Adenosine 5’-diphosphate (ADP)	COVID-19	IVW	25	0.958 (0.920, 0.997)	.035	0.05	14.243 (0.941)	−0.003 (0.677)
Nonadecanoate (19:0)	COVID-19	IVW	24	0.944 (0.892, 0.999)	.044	0.779	16.898 (0.814)	−0.001 (0.882)

Q(*P*), Cochran’s Q test quantified heterogeneity among SNPs, with *P* > .05 indicating no heterogeneity evidence; Egger (*P*), MR-Egger’s and intercept were used to assess potential heterogeneity and horizontal pleiotropy, with *P* > .05 indicating no pleiotropy evidence.

IVW = inverse variance weighted, Nsnp = number of single nucleotide polymorphism, OR = odds ratio, RE_p = reverse MR result.

The sensitivity analysis results showed that the direction of all Beta values across different methods was consistent (see Table S8, Supplemental Digital Content, http://links.lww.com/MD/O434 and Figure S5, Supplemental Digital Content, http://links.lww.com/MD/O435, Table S8, Supplemental Digital Content, http://links.lww.com/MD/O434 illustrates the 5 bidirectional Mendelian randomization models estimate the causal effects of blood metabolites on COVID-19, Figure S5, Supplemental Digital Content, http://links.lww.com/MD/O435 illustrates the Scatter plots of the SNP-blood metabolites and SNP-COVID-19 association estimates for eighteen blood metabolites). However, results were only significant using the IVW method, indicating the findings are relatively unstable. Heterogeneity and pleiotropy tests for the metabolites showed no statistical significance (*P* > .05), suggesting that heterogeneity and pleiotropy do not influence the results (see Table 2 and Figure S6, Supplemental Digital Content, http://links.lww.com/MD/O435, Table 2 illustrates the Bidirectional Mendelian randomization analyses of the causal effects between blood metabolites and COVID-19. Figure S6, Supplemental Digital Content, http://links.lww.com/MD/O435, illustrates the Funnel plots between eighteen blood metabolites and COVID-19 risk estimates). The “leave-one-out” analysis indicated that the effect sizes of the included IVs were close to the total effect size, with no SNPs significantly influencing the causal estimates (see Figure S7, Supplemental Digital Content, http://links.lww.com/MD/O435, which illustrates the Leave-one-out plots showed sensitivity analysis results between eighteen blood metabolites on COVID-19 risk). A forest plot was also used to visually display the estimated effect of each SNP on the influence of blood metabolites on COVID-19 susceptibility (see Figure S8, Supplemental Digital Content, http://links.lww.com/MD/O435, which illustrates the Forest plots showed the association between eighteen blood metabolites and COVID-19 risk under the IVW method).

### 3.4. Mediation analysis of gut microbiota – blood metabolites – COVID-19 susceptibility

**Figure 2.**
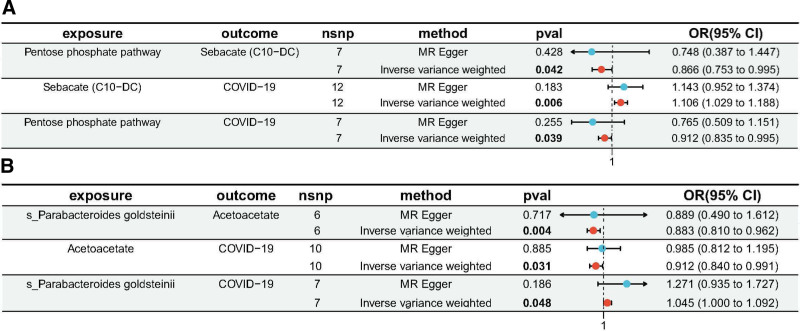
That illustrates the forest plots showed the causal effects of blood metabolites on gut microbiota and COVID-19 susceptibility by using different Mendelian randomization methods. (A) Mediation mode of “Pentose phosphate pathway non oxidative branch – Sebacate (C10-DC) – COVID-19” in two-step Mendelian randomization; (B) mediation mode of “Parabacteroides goldsteinii – Acetoacetate – COVID-19” in two-step Mendelian randomization. CI = confidence interval, nsnp = number of single nucleotide polymorphism, OR = odds ratio.

The results of the TSMR analysis (see Fig. 2, and Table S9, Supplemental Digital Content, http://links.lww.com/MD/O434, which illustrates the 5 Mendelian randomization models estimate the causal effects of blood metabolites on gut microbiotas and COVID-19. Figure 2 illustrates the Forest plots showed the causal effects of blood metabolites on gut microbiota and COVID-19 susceptibility by using different Mendelian randomization methods). indicate that in the first step, which calculates the causal effect of the exposure on the mediator, only 2 out of 34 metabolites with a causal relationship to COVID-19 susceptibility showed a causal relationship with gut microbiota. The IVW results revealed an association between 2 microbial taxa and 2 metabolites. The Pentose phosphate pathway non-oxidative branch was found to reduce Sebacate (C10-DC) levels (OR = 0.866, 95% CI: 0.753–0.995, *P* = .042, FDR = 0.044) (Table S9, Supplemental Digital Content, http://links.lww.com/MD/O434 and Fig. 2A), and the species *P goldsteinii* was negatively associated with Acetoacetate levels (OR = 0.883, 95% CI: 0.810–0.962, *P* = .004, FDR = 0.020) (Table S9, Supplemental Digital Content, http://links.lww.com/MD/O434 and Fig. 2B).

In the second step, which calculates the causal effect of the mediator on the outcome, Sebacate (C10-DC) levels (OR = 1.106, 95% CI: 1.029–1.188, *P* = .006, FDR = 0.046) were negatively associated with COVID-19 susceptibility (Table S9, Supplemental Digital Content, http://links.lww.com/MD/O434 and Fig. 2A), while Acetoacetate levels (OR = 0.912, 95% CI: 0.840–0.991, *P* = .031, FDR = 0.068) were positively associated with COVID-19 susceptibility (Table S9, Supplemental Digital Content, http://links.lww.com/MD/O434 and Fig. 2B).

There was no significant heterogeneity according to the q-statistic for the IVW test and MR-Egger regression (*P* values ranged from 0.132–0.963). The *P* values for the MR-Egger intercept ranged from 0.147 to 0.984, indicating minimal horizontal pleiotropy. Sensitivity analysis results can be found in Table S9, Supplemental Digital Content, http://links.lww.com/MD/O434 and Figures 9–12, Supplemental Digital Content, http://links.lww.com/MD/O435 (see Table S9, Supplemental Digital Content, http://links.lww.com/MD/O434 and Figures 9–12, Supplemental Digital Content, http://links.lww.com/MD/O435, Table S9, Supplemental Digital Content, http://links.lww.com/MD/O434 illustrates the 5 Mendelian randomization models estimate the causal effects of blood metabolites on gut microbiotas and COVID-19. Figure S9, Supplemental Digital Content, http://links.lww.com/MD/O435 illustrates the TSMR Scatter plots showed SNP-gut microbiotas, SNP-blood metabolites and SNP- COVID-19 risk association estimates. Figure S10, Supplemental Digital Content, http://links.lww.com/MD/O435 that illustrates the TSMR Funnel plots between gut microbiotas, blood metabolites, COVID-19 risk estimates. Figure S11, Supplemental Digital Content, http://links.lww.com/MD/O435 that illustrates the TSMR Leave-one-out plots showed sensitivity analysis results between gut microbiotas, blood metabolites and COVID-19 ris. Figure S12, Supplemental Digital Content, http://links.lww.com/MD/O435 that illustrates the TSMR Forest plots showed the association between gut microbiotas, blood metabolites, COVID-19 risk estimates under the IVW method).

**Figure 3.**
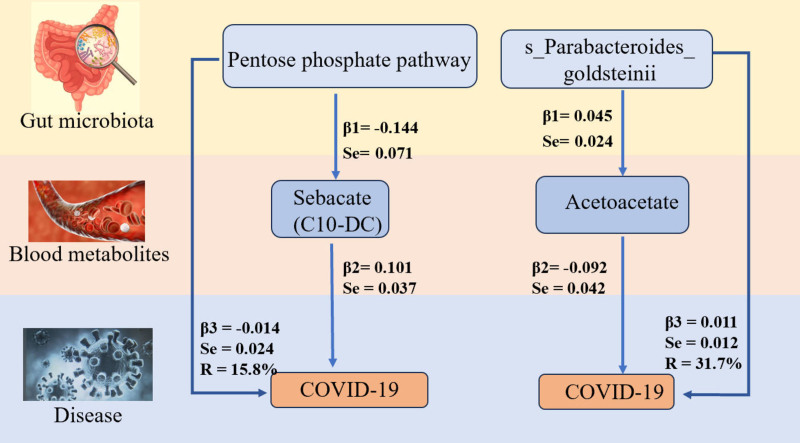
That illustrates the Mendelian randomization analyses show causal effects of blood metabolites on gut microbiota and COVID-19 susceptibility. The diagram displays the mediation mode of “gut microbiota – blood metabolites – COVID-19” in two-step Mendelian randomization. Beta values (β) indicate the causal effect estimates using the inverse-variance-weighted method. R indicate the mediation proportion of mediator.

By calculating the indirect effects mediated by these metabolites and their proportions, we observed that Sebacate (C10-DC) levels had an indirect effect between the abundance of the Pentose phosphate pathway non-oxidative branch and COVID-19 susceptibility, with a mediation proportion of 15.8%. Acetoacetate levels had a mediation proportion of 31.7% between the abundance of the species *P goldsteinii* and COVID-19 susceptibility (see Fig. 3, Which illustrates the Mendelian randomization analyses show causal effects of blood metabolites on gut microbiota and COVID-19 susceptibility).

## 4. Discussion

This study utilized public databases of gut microbiota and COVID-19 susceptibility data, employing a two-sample MR method to explore the causal relationships between 412 gut microbiota species (comprising 207 microbial taxa and 205 functional pathways) and COVID-19 susceptibility. The analysis revealed causal relationships between COVID-19 susceptibility and 3 gut microbiotas. Increased abundance of *P goldsteinii* species was found to elevate the risk of COVID-19 susceptibility, whereas higher abundance of the Pentose phosphate pathway non oxidative branch and L-isoleucine biosynthesis pathway was associated with reduced susceptibility to COVID-19. Currently, there is no direct evidence demonstrating a beneficial causal effect of *P goldsteinii* species on COVID-19 susceptibility. However, literature suggests that increased abundance of microbiota from the Bacteroidetes and Firmicutes phyla is associated with the severity and adverse outcomes of COVID-19, highlighting the need for further investigation into the interaction between *P goldsteinii* species and COVID-19 susceptibility.^[28,29]^ We also identified 2 functional pathways that may reduce the risk of COVID-19 infection, including the L-isoleucine biosynthesis pathway and the Pentose phosphate pathway non-oxidative branch. A previous clinical study found that patients susceptible to COVID-19 exhibited inhibition of short-chain fatty acids and L-isoleucine biosynthesis in their gut microbiota.^[5]^ Another study showed that many amino acids, including isoleucine, were significantly reduced in patients susceptible to COVID-19, indicating disruptions in energy metabolism and amino acid metabolic pathways.^[30]^ Our research found that the abundance of the Pentose phosphate pathway non-oxidative branch was negatively correlated with the risk of COVID-19 susceptibility. Research has suggested that a deficiency in the energy metabolism enzyme G6PD, associated with the Pentose phosphate pathway, can promote SARS-CoV-2 infection.^[31]^ However, whether a reduced abundance of the Pentose phosphate pathway non-oxidative branch can promote SARS-CoV-2 infection requires further investigation.

By using a two-sample Mendelian Randomization (MR) approach to investigate the causal relationship between 1400 blood metabolites (1091 blood metabolites and 309 metabolite ratios) and COVID-19 susceptibility, we found that 32 metabolites and 2 metabolite ratios had a causal relationship with COVID-19 susceptibility. Most of these metabolites originated from amino acid and lipid super-pathways. Among the 18 metabolites in these super-pathways, except for Isobutyrylglycine, 6 metabolites involved in amino acid metabolism were negatively associated with the risk of COVID-19 infection, with Indolelactate levels showing the greatest negative effect. This suggests that various amino acid metabolic pathways are disrupted under conditions of COVID-19 susceptibility, a finding supported by multiple studies. For instance, Nisoli et al^[32]^ measured blood amino acid levels in hospitalized COVID-19 patients and found that levels of 27 amino acids were elevated compared to the control group. Liang et al^[33]^ also noted significant changes in lipid and amino acid metabolism in COVID-19 patients. Among the 8 metabolites involved in the lipid super-pathway, 5 were negatively associated with COVID-19 susceptibility. Notably, Acetoacetate levels, a product of lipid breakdown, showed the most significant negative effect on COVID-19 susceptibility. This association is indirectly supported by a study indicating that Acetoacetate can improve brain glucose metabolism impaired by COVID-19 infection.^[34]^ Our results also indicated that blood metabolism is altered in COVID-19 infection, with Allantoin levels having a positive effect on COVID-19 susceptibility. It is well known that COVID-19 infection is characterized by a high inflammatory state and coagulation disorders, which may be related to purinergic signaling molecules. A study on targeted and untargeted metabolomics found increased levels of adenosine, hypoxanthine, and xanthine in severe COVID-19 patients, which can lead to increased Allantoin levels as the end product.^[35]^

Our mediation MR study provides genetic evidence demonstrating that certain specific blood metabolites mediate the causal effects of gut microbiotas on COVID-19 susceptibility. The study shows that Acetoacetate levels mediate the effect of *P goldsteinii* abundance on COVID-19 susceptibility. Additionally, sebacate (C10-DC) levels mediate the effect of the Pentose phosphate pathway non-oxidative branch on COVID-19 susceptibility. To our knowledge, no previous research has directly linked *P goldsteinii* species with Acetoacetate levels. Current studies on *P goldsteinii* mainly focus on its function in reversing insulin resistance. For instance, oral administration of live *P goldsteinii* has been shown to reduce insulin resistance in high-fat diet-fed mice.^[36]^ Acetoacetate is known to have its production significantly inhibited when insulin resistance is reduced. Research indicates that Acetoacetate supplementation can alleviate COVID-19.^[37]^ Therefore, this study posits that *P goldsteinii* may increase COVID-19 susceptibility by reducing insulin resistance, thereby decreasing Acetoacetate synthesis. Wang D et al also noted that insulin therapy is associated with higher mortality rates in COVID-19 patients with type 2 diabetes, which supports our hypothesis that *P goldsteinii* increases COVID-19 susceptibility by reducing insulin resistance.^[38]^

Additionally, the mediation MR study indicated that the Pentose phosphate pathway non-oxidative branch can reduce susceptibility to COVID-19 by decreasing Sebacate (C10-DC) levels. Research on animals and humans with type 2 diabetes has shown that oral administration of Sebacate (C10-DC) can improve blood glucose control and reduce hepatic gluconeogenesis and glucose output.^[39]^ Other studies have indicated that elevated Sebacate (C10-DC) is associated with reduced carbohydrate energy supply, leading to enhanced fatty acid oxidation metabolism.^[40]^ These studies further support our research findings. At present, several gut microbiota and metabolites have been identified as biomarkers for the severity and prognosis of COVID-19 infection, such as Veillonella in the oral cavity, the synthesize SCFAs and tryptophan.^[41–43]^ We consider the results of this study can provide a theoretical basis for finding biomarkers for COVID-19 susceptibility.

This study aims to minimize the impact of potential confounding factors and ensure the reliability of our findings. First, the GWAS summary data for both exposure and outcome used in this study come from different, non-overlapping cohorts, thereby reducing the potential bias caused by sample overlap. Second, since the dataset is primarily composed of individuals of European descent, the effect of population stratification has been minimized, which helps to reduce the impact of racial differences. To ensure the reliability of our MR results, we conducted a series of sensitivity analyses and performed reverse MR analysis to avoid reverse causality bias. Nevertheless, some residual confounding factors may still influence the results.

This study has several limitations. First, Gender and age are among the most common confounders in epidemiology, but we were unable to perform stratified analysis by gender or age groups using the GWAS summary data to estimate and validate specific causal effects by these factors. Second, the COVID-19 databases used in the study are derived from European populations. Expanding the research to include databases from Asian, African, and other ethnic groups is necessary to obtain more robust evidence. Third, the MR method is a theoretical approach for causal relationship analysis. Further animal experiments are needed to provide deeper validation of the causal relationships between gut microbiota and COVID-19 susceptibility. Fourth, the specific mechanisms by which gut microbiota and COVID-19 infection interact, and whether these interactions are mediated by blood metabolites, remain unclear. Further research is required to validate the MR study results and elucidate these mechanisms.

## 5. Conclusion

In conclusion, the study found Acetoacetate and Sebacate (C10-DC) levels mediate the effect of *P goldsteinii* and Pentose phosphate pathway on the COVID-19 susceptibility, respectively. To our knowledge, this is the first comprehensive study to evaluate the causal relationships between gut microbiota, blood metabolites, and COVID-19 susceptibility. These findings underscore the importance of elucidating the underlying mechanisms linking gut microbiotas and COVID-19 susceptibility. The results offer new insights into microbiome-based interventions for COVID-19 susceptibility and metabolite-targeted therapies.

## Acknowledgments

We thank En-Jun Cui for their valuable guidance in processing the data of this work.

## Author contributions

**Conceptualization:** Yu-Wei Weng, Kui‐Cheng Zheng.

**Data curation:** Xiao-Yan Yao.

**Formal analysis:** Xiao-Yan Yao, Jian-Feng Xie.

**Funding acquisition:** Yan-Hua Zhang, Kui‐Cheng Zheng.

**Investigation:** Yu-Wei Weng.

**Methodology:** Xiao-Yan Yao, Yan-Hua Zhang, Yu-Wei Weng.

**Project administration:** Yan-Hua Zhang, Jian-Feng Xie, Kui‐Cheng Zheng.

**Software:** Xiao-Yan Yao.

**Supervision:** Yu-Wei Weng, Jian-Feng Xie, Kui‐Cheng Zheng.

**Visualization:** Xiao-Yan Yao.

**Writing – original draft:** Xiao-Yan Yao, Yan-Hua Zhang, Yu-Wei Weng.

**Writing – review & editing:** Yan-Hua Zhang, Yu-Wei Weng, Jian-Feng Xie, Kui‐Cheng Zheng.

## Supplementary Material


