# Supplementary material for: Analyzing the causal relationship between gut microbiotas, blood metabolites, and COVID-19 susceptibility: A Mendelian randomization study
Source: Medicine (Baltimore). 2025 Apr 4;104(14):e41445. doi: 10.1097/MD.0000000000041445 (PMC11977742; doi:10.1097/MD.0000000000041445)

Supplemental Figure 1 that illustrates the Scatter plots of the SNP- gut microbiotas and SNP-COVID-19 association estimates for three gut microbiotas.

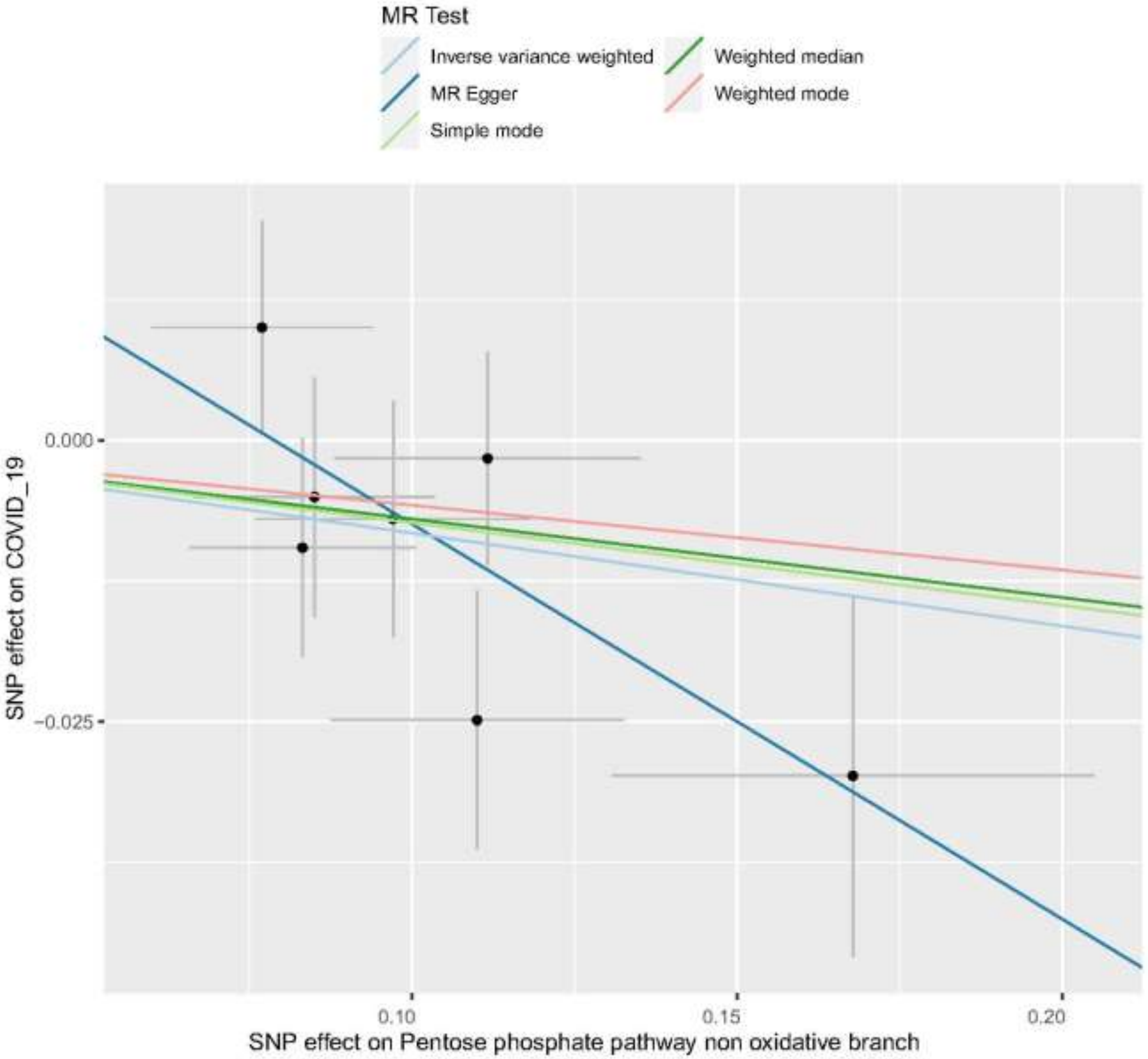

Supplemental Figure 2 that illustrates the Funnel plots between three gut microbiota and COVID_19 risk estimates.

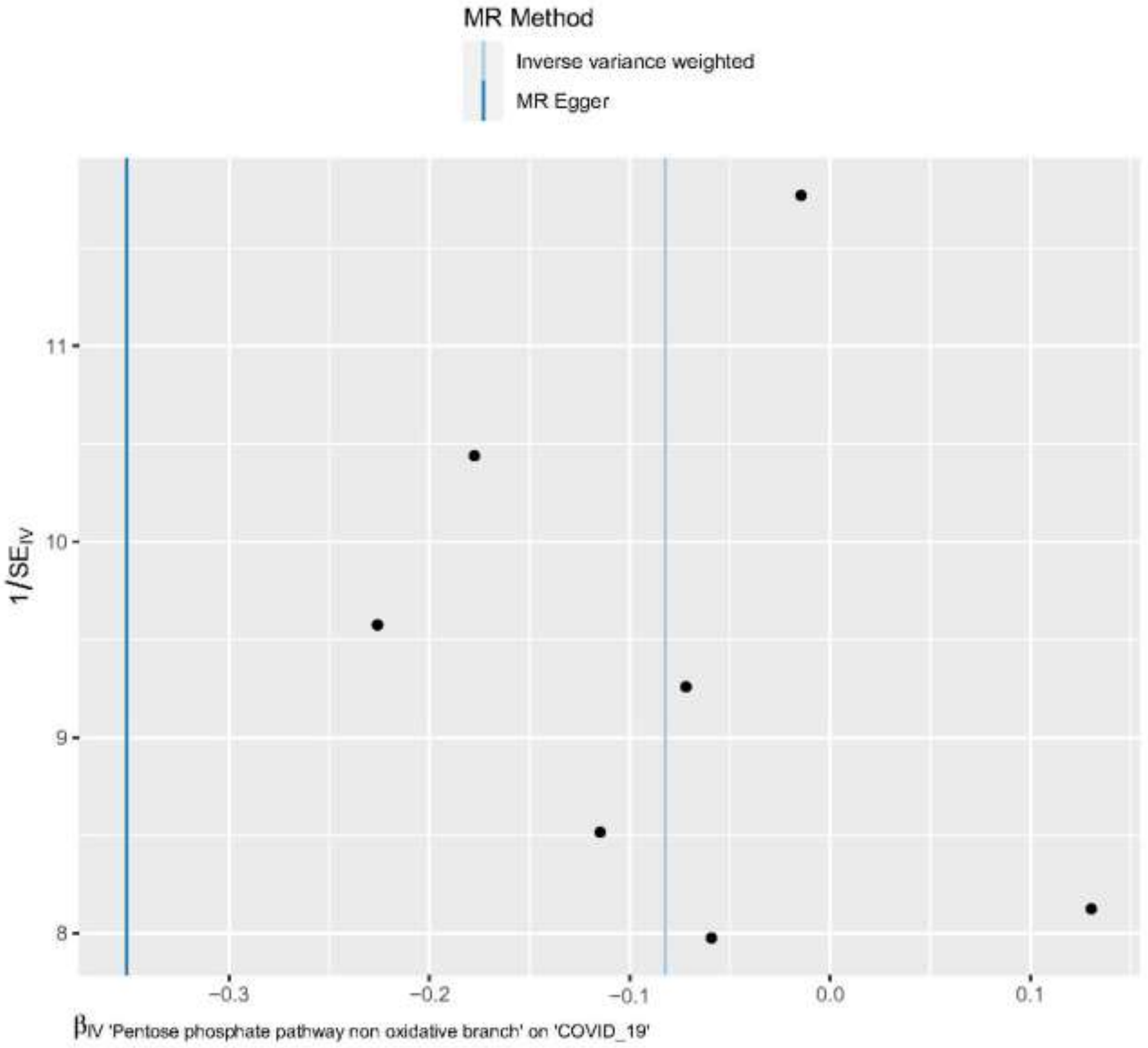

Supplemental Figure 3 that illustrates the Leave-one-out plots showed sensitivity analysis results between three gut microbiotas and COVID-19 risk estimates.

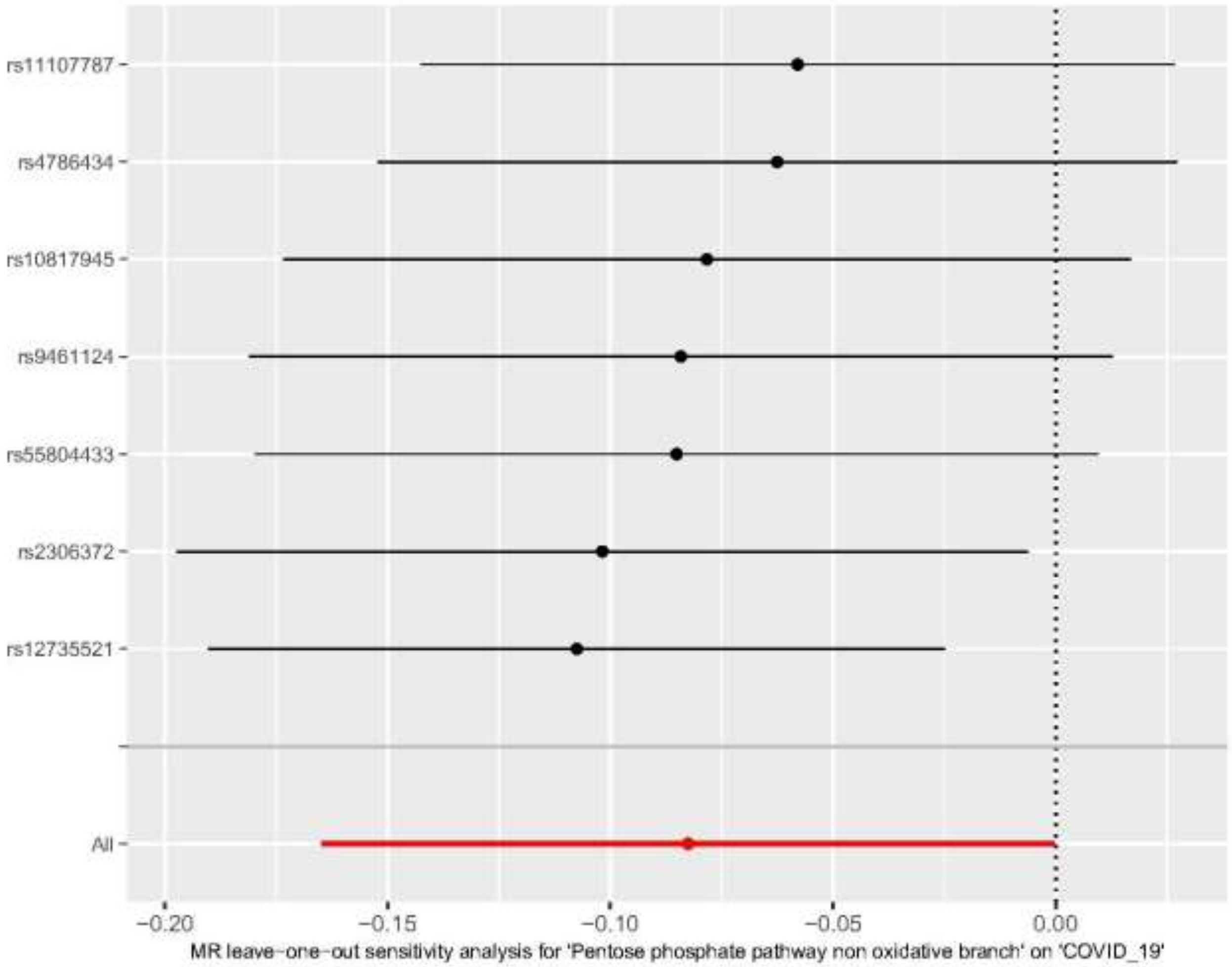

Supplemental Figure 4 that illustrates the Forest plots showed the association between three gut microbiotas and COVID-19 risk under the IVW method.

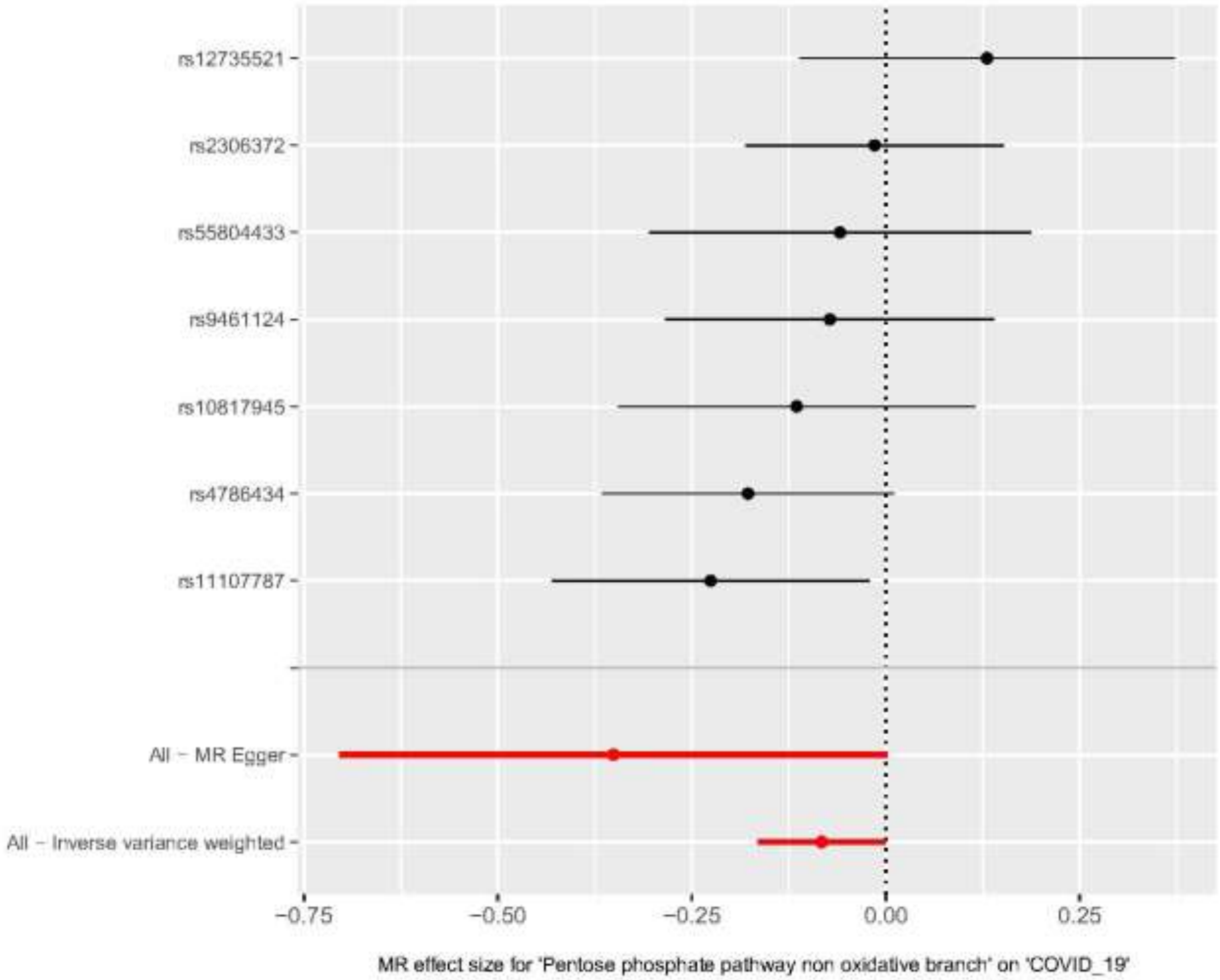

Supplemental Figure 5 that illustrates the Scatter plots of the SNP- blood metabolites and SNP-COVID-19 association estimates for eighteen blood metabolites.

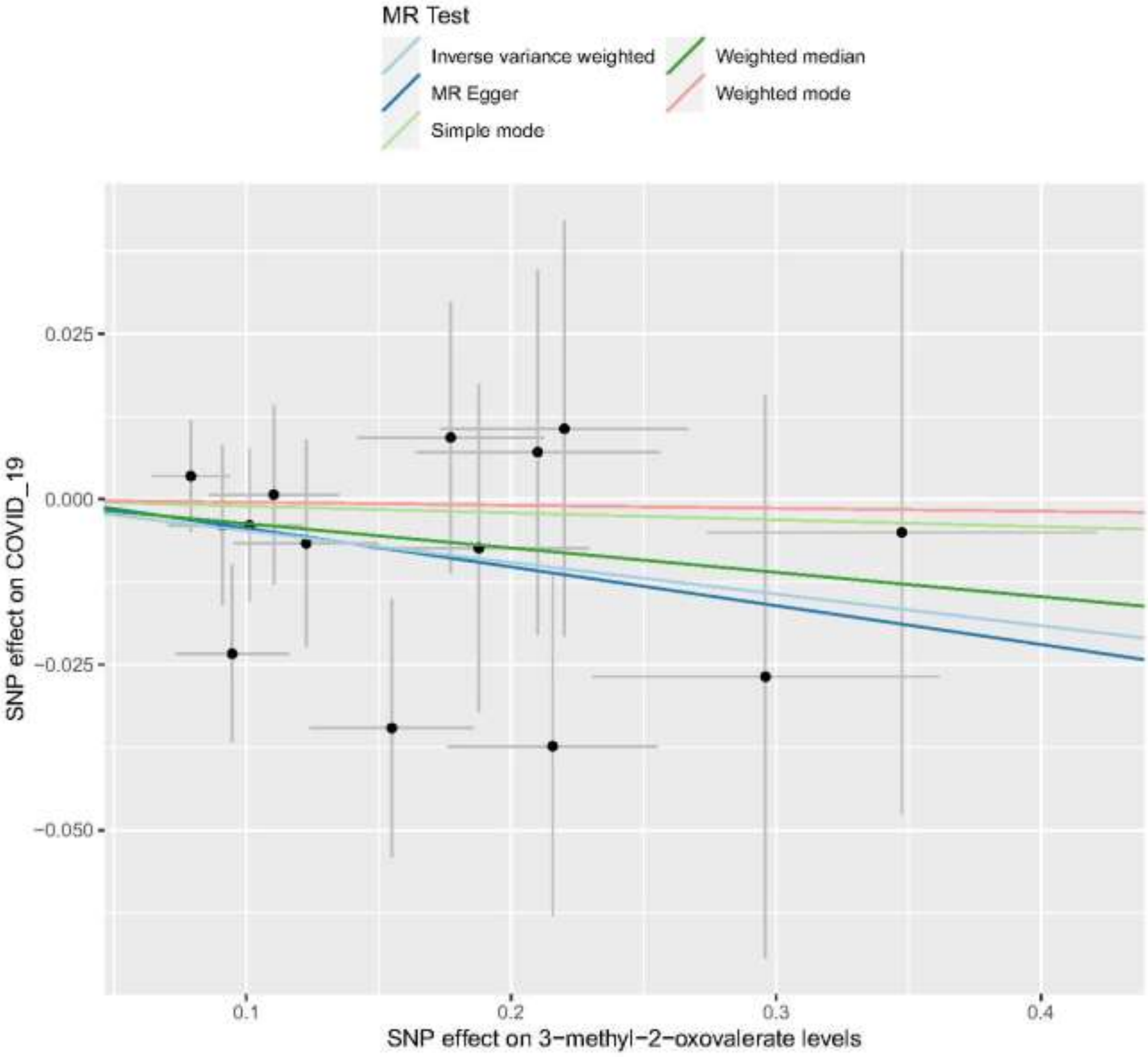

Supplemental Figure 6 that illustrates the Funnel plots between eighteen blood metabolites and COVID-19 risk estimates.

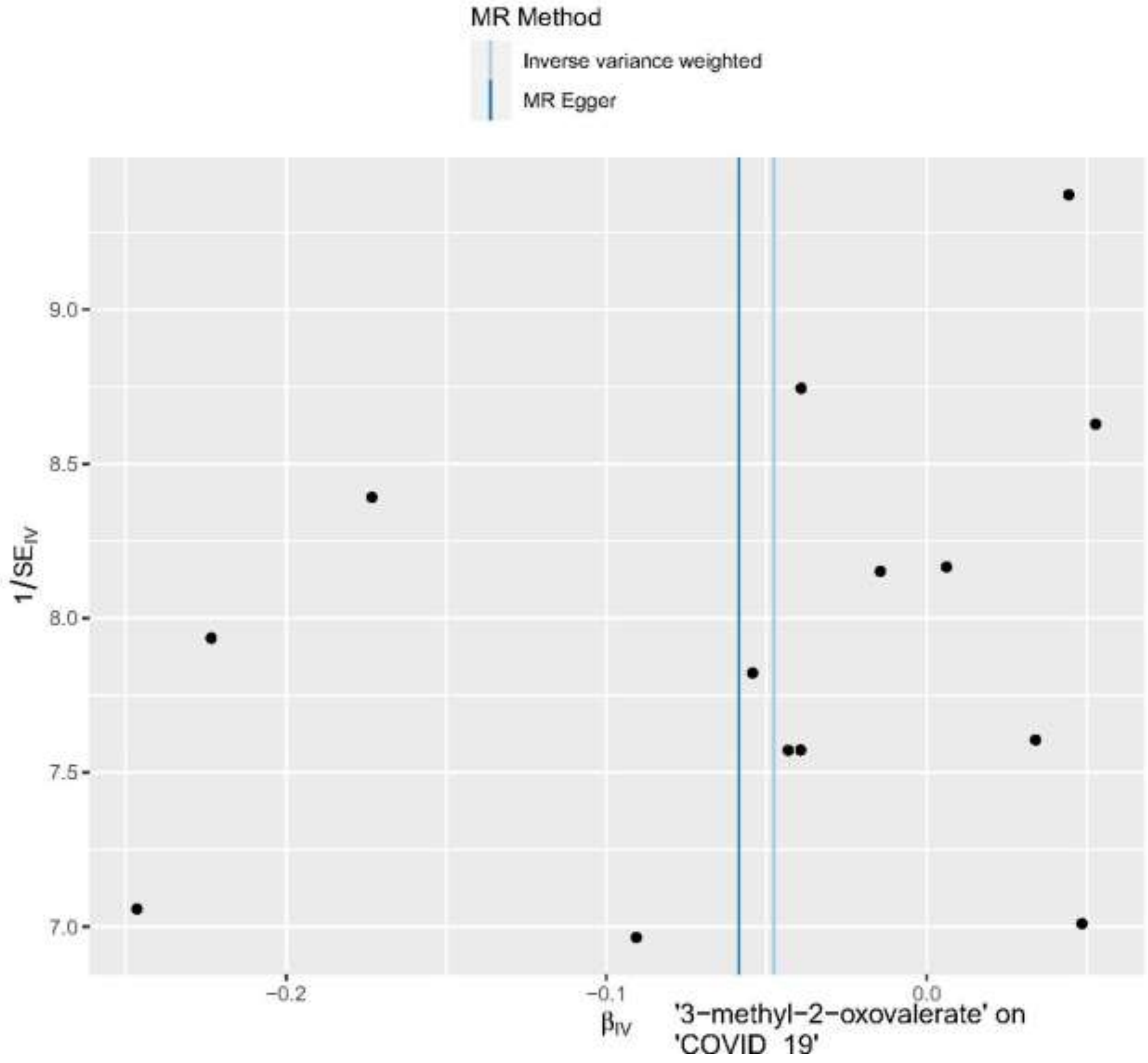

Supplemental Figure 7 that illustrates the Leave-one-out plots showed sensitivity analysis results between eighteen blood metabolites on COVID-19 risk.

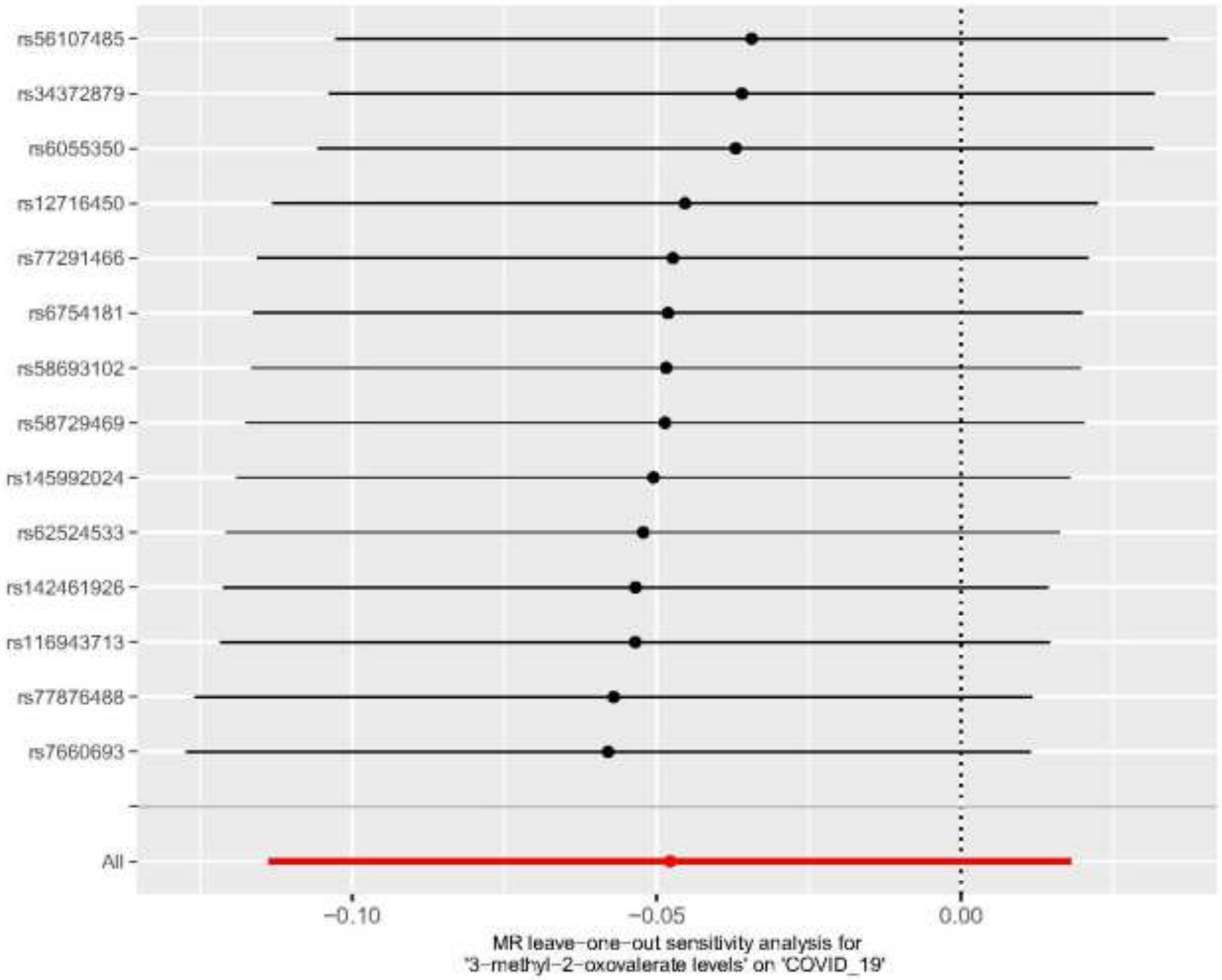

Supplemental Figure 8 that illustrates the Forest plots showed the association between eighteen blood metabolites and COVID-19 risk under the IVW method.

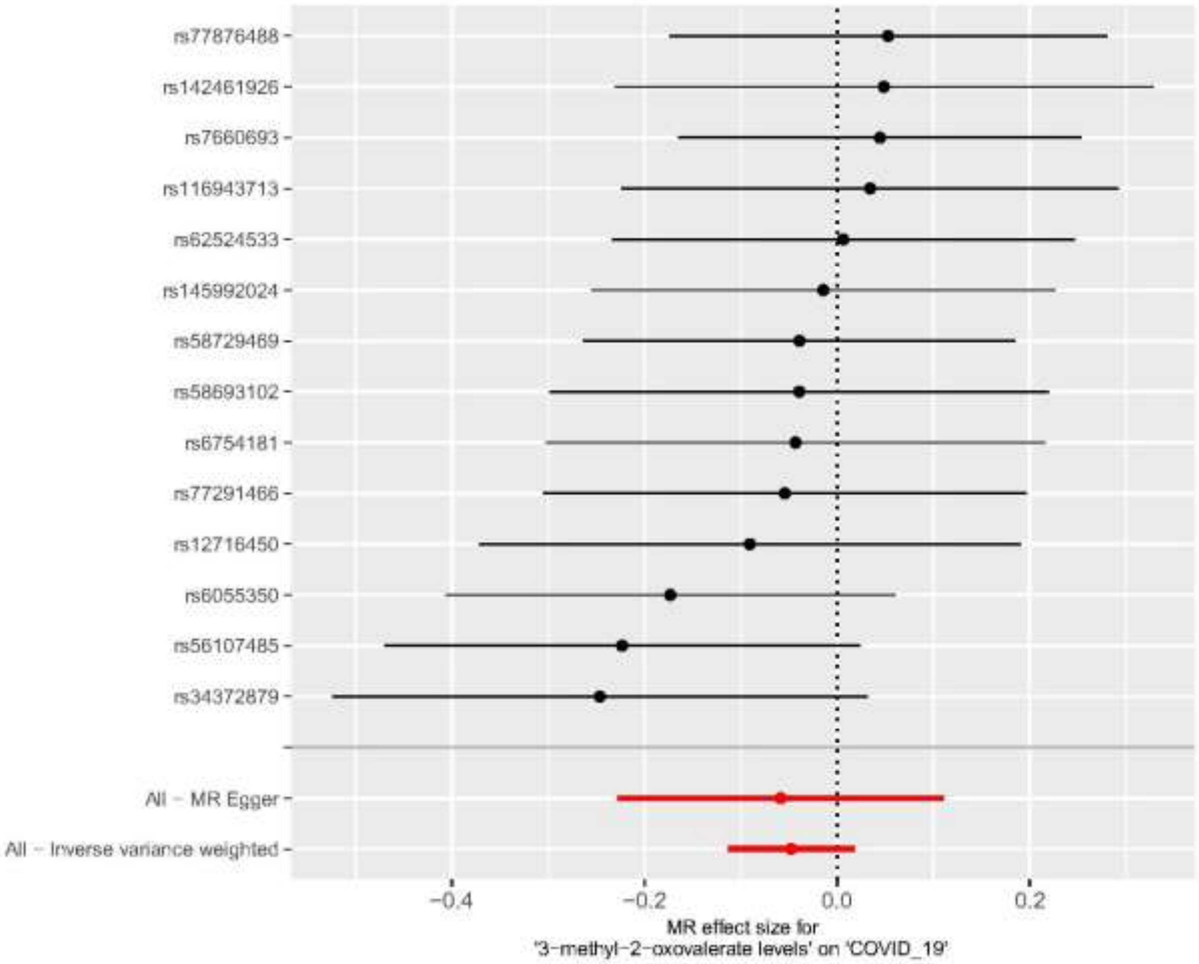

Supplemental Figure 9 that illustrates the TSMR Scatter plots showed SNP- gut microbiotas, SNP- blood metabolites and SNP- COVID-19 risk association estimates.

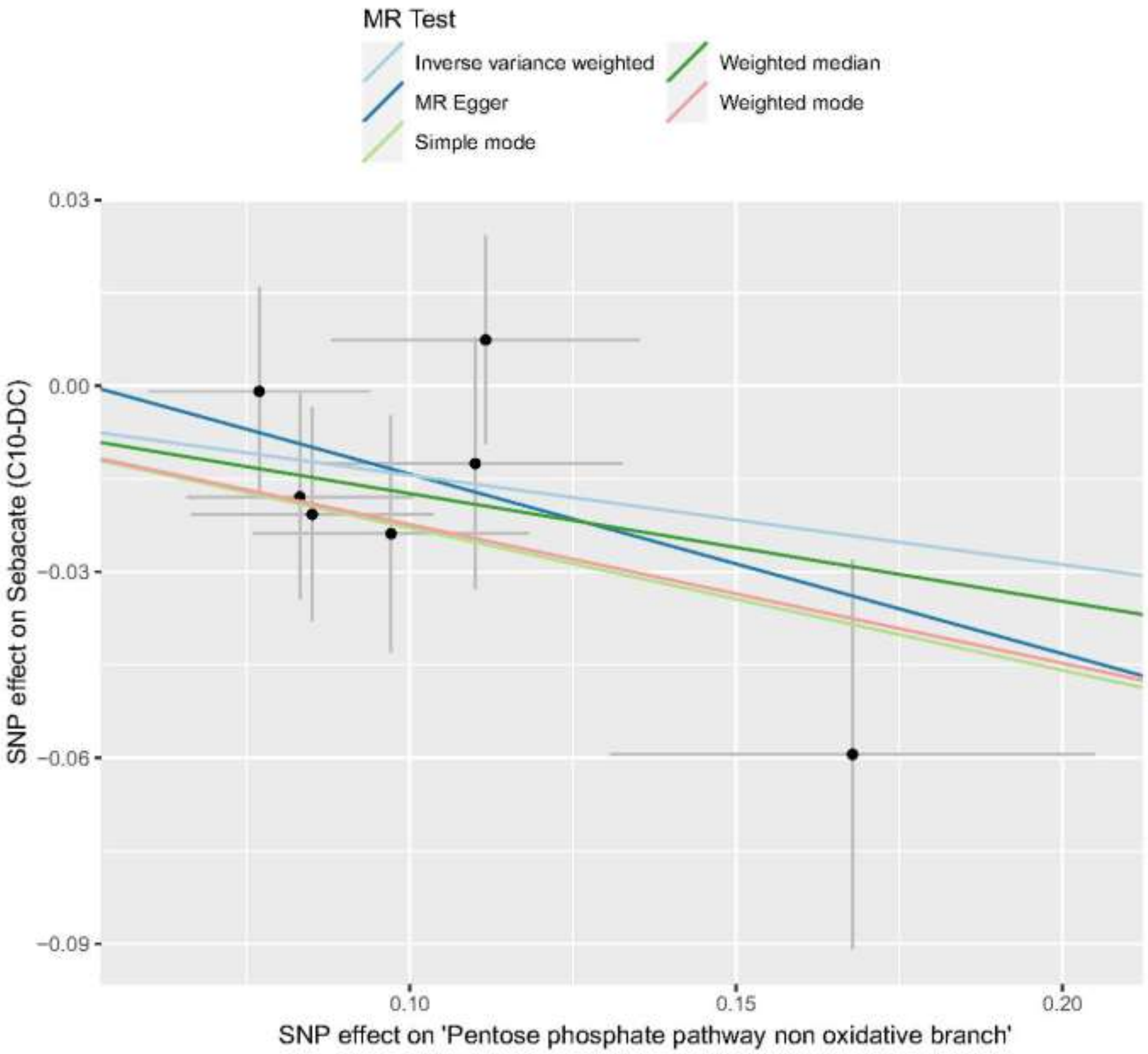

Supplemental Figure 10 that illustrates the TSMR Funnel plots between gut microbiotas, blood metabolites, COVID-19 risk estimates.

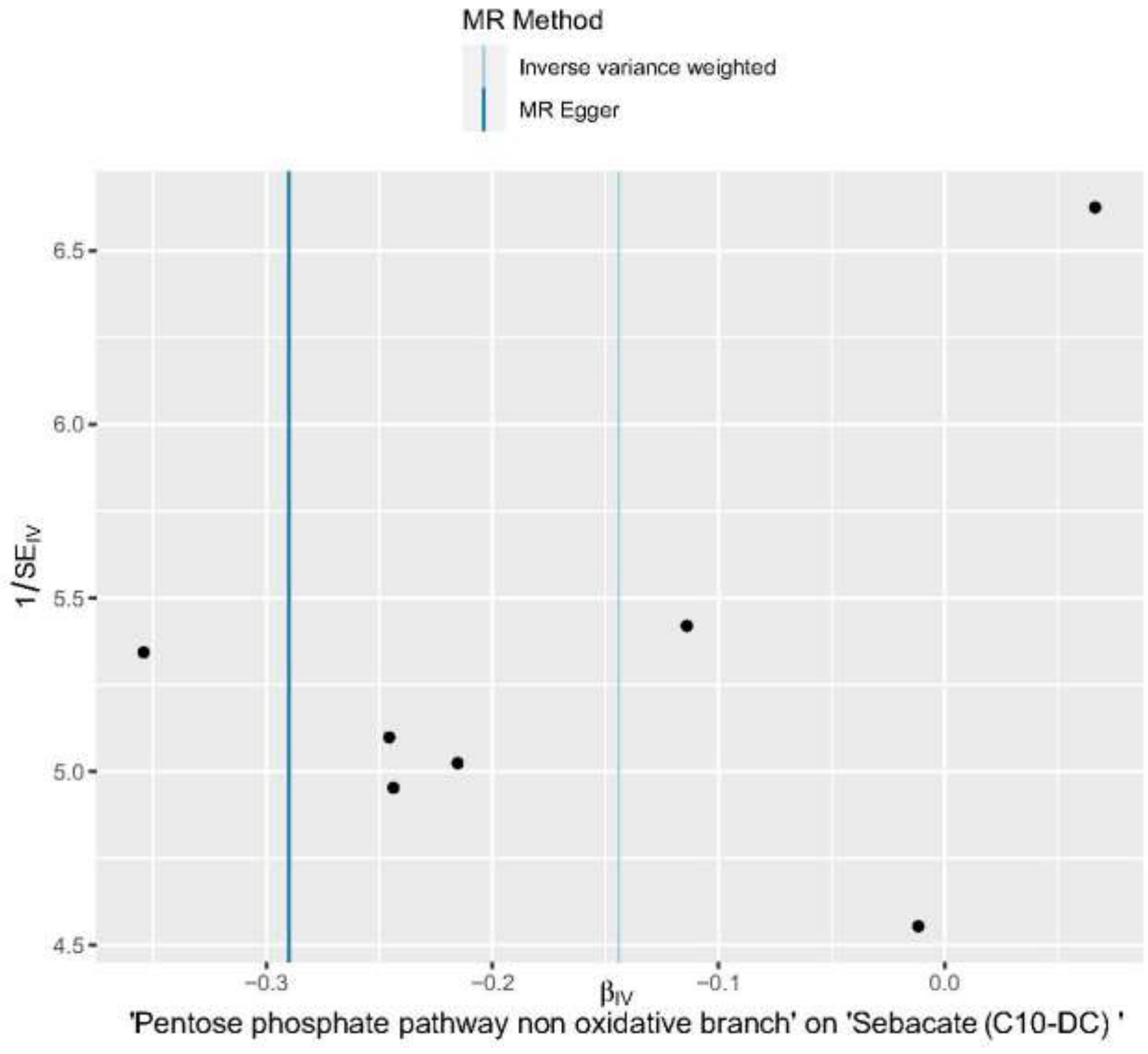

Supplemental Figure 11 that illustrates the TSMR Leave-one-out plots showed sensitivity analysis results between gut microbiotas, blood metabolites and COVID-19 risk.

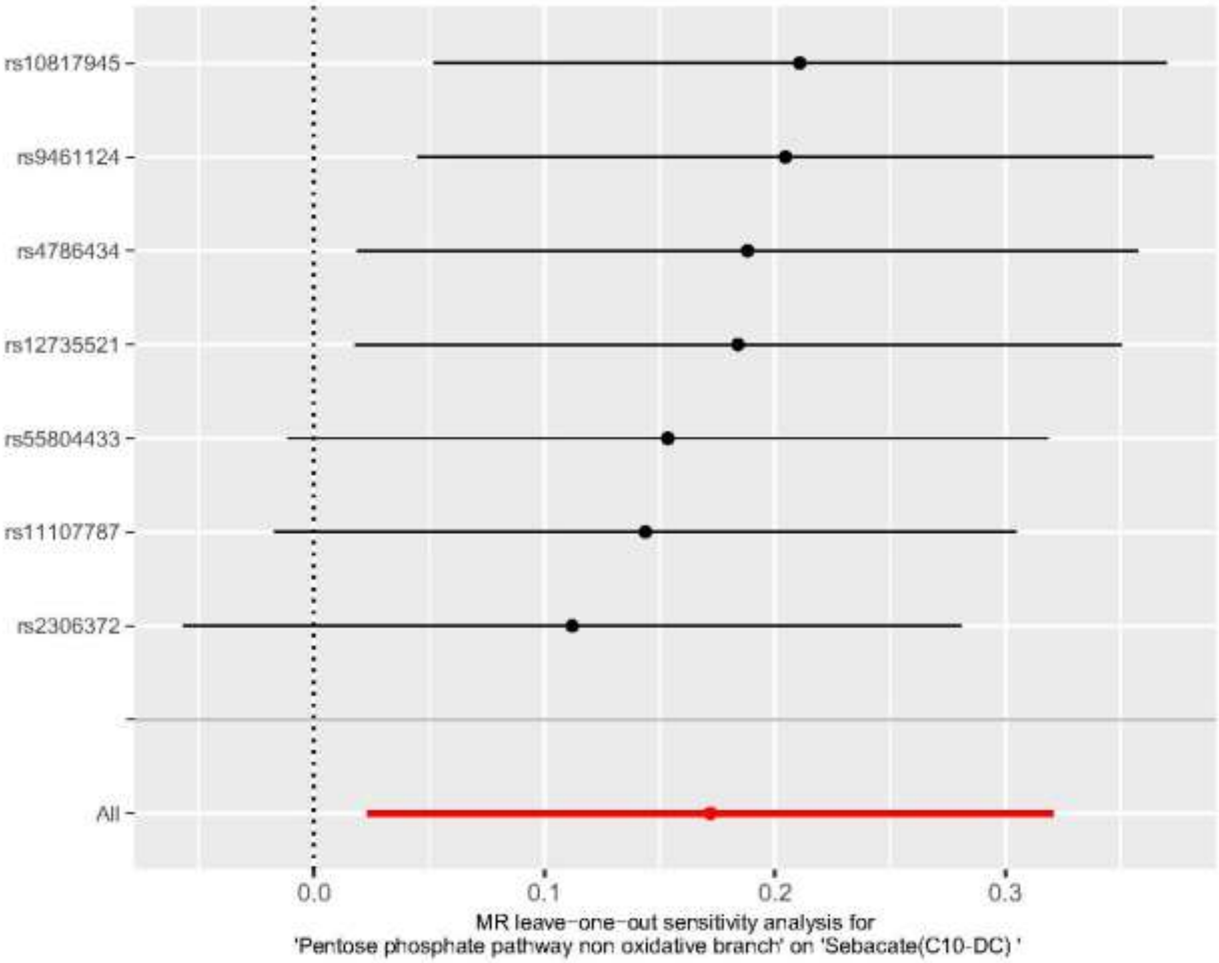

Supplemental Figure 12 that illustrates the TSMR Forest plots showed the association between gut microbiotas, blood metabolites, COVID_19 risk estimates under the IVW method.

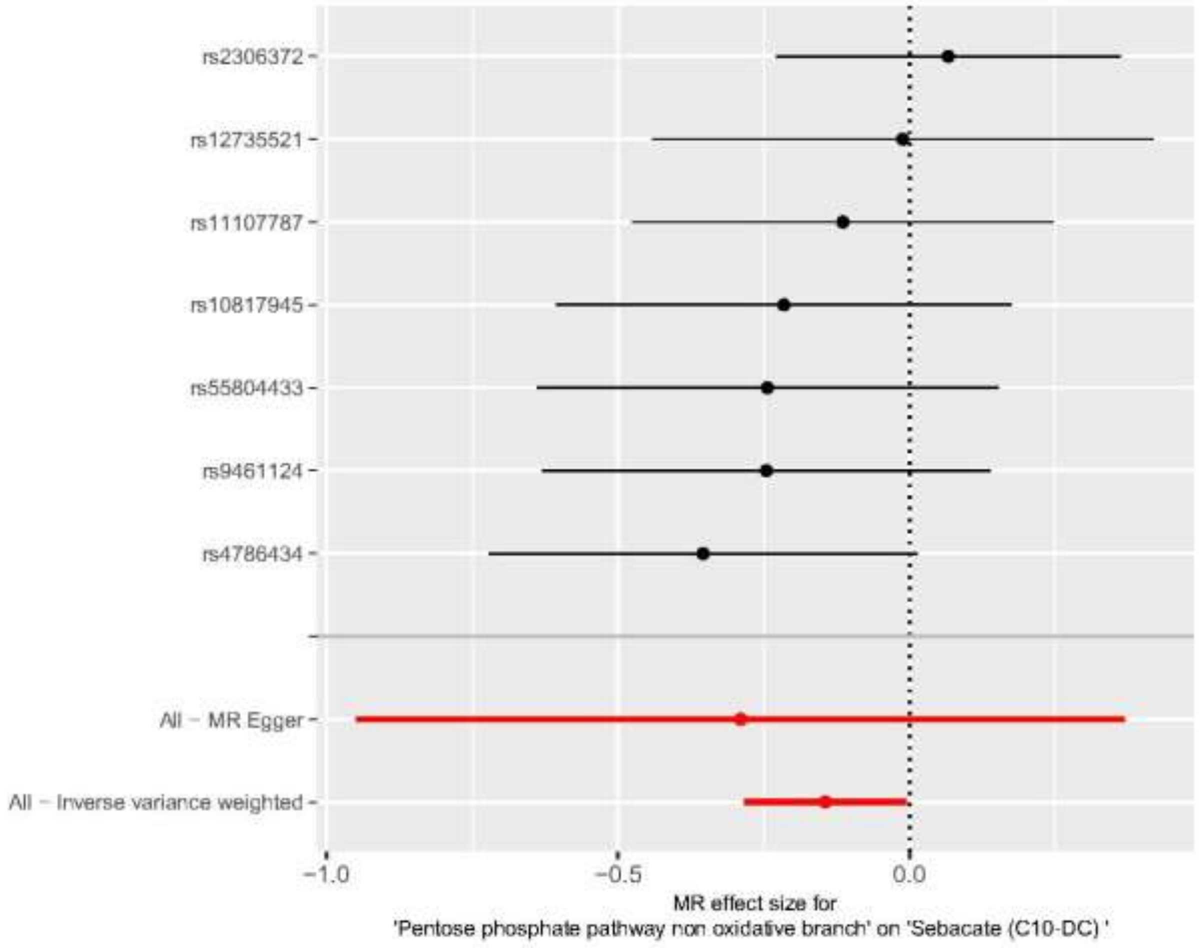

Supplement: Supplementary file 2 [file medi-104-e41445-s002.pdf]
